# Coarse-grained and all-atom MD simulations with Gromacs based on CELLmicrocosmos 2.2 model membranes

**DOI:** 10.1186/1758-2946-3-S1-P43

**Published:** 2011-04-19

**Authors:** B Sommer, T Dingersen, C Gamroth, AJ Heissmann, G Lukat, R Rotzoll, S Rubert, A Schäfer, J Krüger

**Affiliations:** 1 Bio-/Medical Informatics Department, Bielefeld University, 33615 Bielefeld, Germany; 2 Department of Chemistry, University of Paderborn, 33098 Paderborn, Germany

## 

The CELLmicrocosmos MembraneEditor (CmME) [1] enables researchers to generate PDB [2] based membrane structures in a convenient way. The lipid distribution is computed by algorithms working on the outer shapes of the molecules. For this reason, the computation and visualization process is very fast, while the atomistic structure of each single molecule remains unchanged.

PDB membranes can be exported to Gromacs [3], a molecular dynamic simulation (MD) program. In this new approach the workflow between CmME and Gromacs has been improved. Two major strategies for the simulation of membranes are the all atom (AA) and coarse-grained (CG) approach. As a logical consequence of the shape-based principle of CmME, coarse-grained support has been implemented. The ongoing work is presented by comparing AA and CG structures generated with CmME, simulated with Gromacs and reverse-parsed to CmME.

Newly implemented features of the CmME MD Edition:

• The Membrane Shifter Tool, enabling membrane model repositioning

• Shape generation taking periodic boundaries into account

• A Molecule Editor, supporting the definition of CG particles

• Advanced raft support, allowing the analysis of lipid values and raft-restricted computation of lipid distributions.

In addition, a GUI based on the CmME algorithm interface is being implemented, allowing the comfortable handling of CmME and Gromacs based workflows.
